# Proceedings and Discussions of the Twenty-fourth Annual Meeting of the American Dental Association

**Published:** 1884-10

**Authors:** E. G. Betty


### PROCEEDINGS.

### Proceedings and Discussions of the Twenty-Fourth Annual Meeting of the American Dental Association.

REPORTED BY E. G. BETTY, D.D.S.

The twenty-fourth annual meeting of this body took place at Saratoga Springs, N. Y., August 5th, of the current year, in the Town Hall. President Darby called the members and delegates to order at 11 o'clock, when prayer was offered by the Rev. Dr. Joseph Carey, of Saratoga, rector of the Bethesda Episcopal Church. The Treasurer, Dr. Geo. Keely, then called the roll, and a goodly number answered to their names. The reading of the minutes of last years's meeting was dispensed with on motion of Dr. McKellops. The report of the Committee on Arrangements made its report per Dr. Odell, Chairman, in which the hours of the daily sessions were fixed at from 9 30 a.m. to 2 p.m., and from 8 p.m. to adjournment. The report also included the hours at which the several sections were to meet, etc. On motion of Dr. Pierce the report was adopted. Dr. Odell moved that Wednesday afternoon, from 3 to 7 o'clock be devoted to clinics. Dr. Allport thought that it was better not to take action on the subject until it was known what papers there might be presented for reading, as they have the preference over other business; he withdrew his motion upon learning that the order of business had made provision for the holding of clinics; the motion was carried. Dr. Crouse, Chairman of the Committee on Credentials, then read the list of delegates in attendance. The Secretary, Dr. Cushing, stated that he had been requested by many members to offer an amendment to the constitution (which could be done by unanimous consent) referring to the working of the sections, as the failure to accomplish their work was due to their organization being postponed till so late in the meeting that there were not enough members present to elect officers. The amendment was to put the time earlier in the meeting and also to partly change the method of working. On motion the change, or amendment rather, was adopted unanimously. President Darby then read his address, in which appears the following :

---

*Gentlemen of the American Dental Association—*

One quarter of a century ago a company of gentlemen met at Niagara Falls and formed an association that has continued to meet, with one exception, every year since. Their object was to promote the interests of the science of dentistry with all that implies as well as to promote a friendly and social feeling among the members of the profession. Some of the original members are still living, others have passed through the portals of death. In this connection it becomes my sad duty to announce the death of Prof. Buckingham, a man in every sense of the word. During the last quarter of a century, the infant child has become a stalwart man. Since that time the population of the United States has increased from 30,000,000 to 57,000,000; a great war has been fought, great industries have been developed, and great discoveries made, such as the telephone, phonograph, etc. In 1859 the number of dentists engaged in the work of their profession was placed at 4,000; to-day there are 12,000. Societies have multiplied in like proportion as have our schools, periodicals, enactment of laws, etc. In admitting candidates to our professional schools there must be preliminary examination in order that those only, who are qualified, can be permitted to join the ranks. The dental schools of the United States need endowments most, so that the professors engaged in the work of education may be enabled to devote their entire time and energy to that purpose. If each dentist in the United States would give $5.00 per annum for five years, we could establish and maintain one or more such colleges. The present tendency seems to be the adding of dental departments to medical schools; this has both its merits and demerits. In the dental colleges the professors have worked hard and long, a labor of love, often with inadequate compensation, often with none. The meeting of the College Faculties just held, it is hoped, will establish and maintain a uniform standard, etc., and that a year hence will see its good fruits. The State examining boards should make their examinations as rigid as those of the College

Faculties so that improper candidates cannot practice. Another evidence of our progress is the improvement in the quality of our literature. In the last decade the work done as published in our journals shows an equal ability with foreign investigators. New text books appear every now and then, and evidence is everywhere shown of our influence and progress.

This Association has not always received hearty support as seen by the fickle and uncertain attendance upon its deliberations. This too in spite of the fact that its policy is liberal, the conditions of membership not unreasonable, and it always receives duly accredited delegates from other societies.

Fault has been found with its method of permanent membership and any action looking towards a change in it can only be determined by experiment. There may be other causes of the failure of the Association to accomplish all that was expected of it. It may rest in the body of the profession itself, the members of which do not attend regularly and many who do attend, do not participate in its proceedings. The needs of the Association can be embraced in one sentence, " more honest work and less adverse criticism."

At the conclusion of the address, Dr. J. G. Ambler moved that a committee be appointed to draught suitable resolutions on the death of Prof. Buckingham; carried. Dr. McKellops moved that a committee of five be appointed to consider that portion of the President's address relating to the appointment of delegates, the committee to consist of Drs. E. Parmley Brown, J. Taft, A. W. Harlan, Foster, and McElhaney; carried. Dr. Cushing, Chairman of the Committee on Publication, reported that the committee had made the same arrangements as formerly with the S. S. W. Co., for the publication of the transactions of the Association. The report was accepted and referred to the Finance Committee. Dr. Stockton now moved that the President's whole address, excepting that portion relating to Prof. Buckingham, be referred to McKellops' committee. Some discussion then ensued as to the time of the meeting of sections; Dr. Taft moved that this session adjourn at 1 o'clock p. m., in order to provide the necessary time. Carried. The names of the members of the several sections were then read and the hours of meeting determined. Dr. Cook stated that there were names in the lists read, who were not members of the Association, and moved that they be dropped. Dr. Frederichs said that names were only used while the persons were members, and if they did not conform to the rules they were subsequently dropped. The Secretary, Dr. Cushing, stated that he was instructed to use all names that did not respond when the sections were announced. Dr. Atkinson then rose and said that when section 3 was read, his name did not appear and he wanted to know the reason why.

*Dr. Cushing.* I must have made a mistake; it was an oversight merely and not intentional.

*Dr. Atkinson.* Am I on *any* committee?

*Dr. Cushing.* I cannot find your name anywhere.

*Dr. Spaulding.* Dr. Atkinson is a whole section by himself: (laughter.)

*Dr. Atkinson.* Then like Dundreary's bird, I will flock all by myself." Adjourned till 8 p. m.

FIRST DAY—EVENING SESSION.

The meeting having been called to order, Pres. Darby announced that he had appointed Drs. J. G. Ambler, Jno. Allen and J. Taft as a committee on memorial resolutions. Dr. Odell stated that section 5 had an interesting paper to report, which would be illustrated by stereopticon views, and moved that Wednesday evening be devoted to its reading.

*Dr. Barrett.* It seems to me that this matter should come from section 5 when its time arrives.

*Dr. Odell.* The reason I moved that it be made a special order, under the head of miscellaneous business, is that, as the Association begins with section 6, it would be entirely too late by the time section 5 would be reached.

*Dr. Crouse.* The gentleman has made every preparation for the exhibition and has gone to the expense of employing men to manage the light, and in view of the treat that is in store for us, I hope the motion will prevail.

*Dr. Barrett.* I insist that the Association should not take the matter from the section, but am satisfied, if it is presented through the proper channel.

*Dr. Rhein.* An evening is necessary for the proper presentation of the paper and its illustrations, for, under the present order of business it will not be reached until Friday morning.

*Dr. Spaulding.* I move that the rules be suspended and that Wednesday evening be devoted to the purpose.

*Dr. Crouse.* It is not necessary to suspend the rules, as the time can be given by making the paper a special order of business.

*Dr. Barrett.* Is this from the section?

*Chair.* It is.

*Dr. Allport.* Let the gentleman read his paper so that we may know what it is.

*Dr. Rhein.* It is on certain disputed points in histology and pathology of the teeth, and the paper is presented by Dr. J. L. Williams, of New Haven, Conn.

Question was called and the motion prevailed. Dr. Taft on the committee, suggested by Dr. McKellops, reported progress and requested that the committee be granted further time. The committee on the memorial resolutions were not ready to report. Dr. A. W. Harlan, Chairman of section 6, read a paper of his own which he said was in the nature of a continuation of the report of last year, and of which the following is a synopsis:

### PYORRHŒA ALVEOLARIS.

The many letters of inquiry and innumerable questions upon this subject, evidencing a wide spread interest in it, have induced me to continue its investigation by experiment and otherwise. The mass of the profession are apt to confound salivary deposits on the lingual and other surfaces of lower incisors and such deposits on the buccal, palatal, or lingual surfaces of molars, for Pyorrhœa Alveolaris. In many cases there is but a slight recession of the gums surrounding the affected teeth, in others, none at all. Between the molars and bicuspids the gums are especially liable to bleed. Wherever the process is thin in its normal condition, there will be found the greatest loss of bony tissue. Pyorrhœa Alveolaris is not a disease of old age only, as it is often found in young patients. The majority of cases occur between the ages of 25 years and 45 years, but as to this fact there is no reliable data to prove it. The future must furnish the statistics upon which to determine the influence of age, sex, temperament, etc. The teeth of "mouth-breathers" are most liable to be affected by the disease. Various deviations of position of the teeth from the normal are observed. The vast majority of cases of Pyorrhœa Alveolaris are unaccompanied with Salivary Calculus. The dentist is often deceived by the appearance of the gums as they do not always exhibit traces of inflammation; few cases will be seen where the *ligamentum dentinum* has completely lost its hold upon the neck of the tooth. I will have something to say about the causes at some other time.

TREATMENT, INSTRUMENTS, ETC.

The instruments that are very necessary to have, are three very delicate probes; their uses are first to determine the depth of the pockets; second, the detection of serumal deposits on the roots; third, to discover the condition of the edge of the alveoli.

It is best when instituting treatment to wash the parts thoroughly first and then dry with paper. Should there be any accumulation of Salivary Calculus it must be removed and the patient furnished with the following prescription:

℞ Pinus Canadensis, alb........... ℥ s s.
Aquae Rosae.....................ʒ iii. ss.
Eugenol..........................*m.* xxx.

M ft. Sig. Use three times a day with a badger hair toothbrush.

This preparation is to be continued for a period of six days.

The removal of serumal deposits should be done thoroughly, and one tooth at a time, several sittings being devoted to it. I use Cushing's scalers for the purpose and find them very efficient. Another very necessary instrument is a good hypodermic syringe, supplied with several different points, one or more of which should be designed for the injection of chemicals exclusively. The Per Oxide of Hydrogen ($H_2O_2$) is to be injected into the pockets immediately after the removal of the deposits. Following which I almost exclusively inject a solution of Iodide of Zinc, 48 grs. to the ounce of water. I have also been using Eugenol and Sanitas Oil, much diluted with Oil of Wintergreen or Sassafras, instead of the Iodide of Zinc, but am not yet ready to report upon their efficiency.

The patient must not use a stiff brush nor irritate the gums by picking the teeth. The use of the Pinus Canadensis is to be discontinued when the pockets are systematically treated as just described. Loose teeth are to be held firm by ligating them with suitable materials. Discontinue the injection of the Hydrogen Per Oxide when pus ceases to discharge. The patient may then be given the following prescription:

℞ Cretae Precip.

Pulv. Iris Florintina......................aa ʒ ii.

Saponis, Castile alb.

Pulv. Borax...............................aa ʒ ss.

Pulv. Gum Myrrha..........................ʒ ii

Honey and Glycerine, q. s. to make soft paste.

The paste may be colored pink if so desired.

After this course of treatment the periods between visits to the dentist may gradually be lengthened. When there is pain accompanying acute cases, Iodoform mixed with a paste with Oil of Cinnamon until the mass is of a consistency of thick cream, is an excellent anaesthetic. The mouth must, of course, be kept scrupulously clean throughout after the treatment.

### ENGENOL AND SANITAS.

Eugenol is one of the active principles of Oleum Carophyllum, and is known as Eugenic acid. It is prepared by decomposing Eugenate of potassium with Sulphuric acid and is afterwards rectified, when a colorless oil of 1076 s. g. is obtained. It has the odor of oil of cloves intensified. It may be diluted with either water or alcohol though its effects as a parasiticide are best obtained in an aqueous solution.

It is used to inject into alveolar abscesses, and after the removal of a recently destroyed pulp, with marked success. It will and should supersede all of the powerful albumen coagulators. The root must always be well filled, and after the injection of it and filling of the root, no further treatment than this is required. It is a powerful germicide, and not dangerous to human life.

SANITAS is obtained by the oxidation of oil of turpentine; it is prepared by directing a current of heated air on its surface. It contains a substance identical with $H_2O_2$, camphoric oxide, and $H_2O_2$ also. It is an oily substance, possessing oxidizing powers equal to a ten-volume solution of $H_2O_2$, and it may be considered as a convenient form of storing $H_2O_2$, as we have in this product a germicide of the first rank, combined with a volatile oily dressing, which is disinfectant, antiseptic, and not poisonous; it is also non-irritating. It is used for injecting the pockets in Pyorrhœa Alveolaris, alveolar abscesses, forms one of the ingredients in tooth-washes, is a dressing for foul ulcers and exposed pulps, and is used in the antrum when occasion requires. In several cases in which I have used it, it has given very satisfactory results. The drug is manufactured by the Sanitas Co., of London, England, and can be procured from Sargent, of Chicago, who imports both this and Eugenol."

*Dr. Odell.* The paper just read by Dr. Harlan I should call just simply lovely. There is but one point in it to which I can take exception, and that is he is adverse to any manipulation of the gums, trusting to the drugs to accomplish the desired result. In my estimation, the manipulation of the gums at a certain stage of the disease is the only thing that will stimulate and keep up the reparative process in the tissues. This can and may be supplemented by the application of the galvanic current; under this treatment cases may be cured. The conclusion to be drawn from this is, that in some cases it is a sort of neurosis, and that electricity will greatly benefit the curative process.

*Dr. Bogue.* I would like to ask Dr. Harlan why it is that at one stage of the disease he recommends the use of Canada Balsam, and why that, at a certain stage, he advises prepared chalk and myrrh?

*Dr. Harlan.* In the paper it was stated that the prescription was prepared for the patient after the removal of Salivary Calculus, and that it was not given for the treatment of Pyorrhœa Alveolaris. With reference to the myrrh, the quantity prescribed is so small that it might be left out altogether, though it can be used to disguise the taste of the paste; the object was to form a paste instead of a powder.

*Dr. Bogue.* It seems to me that in the treatment of these cases it is necessary to remove all deposits before using medicaments. It is inconsistent to prescribe anything that will produce deposits, as both the substances I mentioned are precipitated by water. I have noticed for many years past, that it is the custom of dentists to prescribe tincture of myrrh, but I am at a loss to understand what it is for.

*Dr. Harlan.* I want to ask Dr. Bogue if he ever uses a powder or paste containing myrrh as one of its ingredients?

*Dr. Bogue.* No; I use glycerine instead.

*Dr. Harlan.* I do, myself, and am not conscious of any deposit resulting from it. If the myrrh is not a good thing in the paste, it may be left out.

*Dr. Barrett.* With the single exception of dental caries, there is no disease so frequently presented to the dentist for treatment as Pyorrhœa Alveolaris. I have long been waiting, and am still waiting, to hear a paper on the etiology of this disease, and until we know the precise causes of the disease, we cannot hope to treat it intelligently and successfully. If it is an affection of the peridental membrane, a certain course of treatment is indicated to combat it; if it is simply a peculiar deposit upon the teeth, then another line of treatment must be pursued. In my experience, the worst cases are those affecting the border of the alveoli, and I have found that scraping it, and the application of astringent and caustic medicaments have not reached the bottom of the trouble. I believe that it is a caries of the margin of the alveolar ridge. It is impossible to secure success as the result of treatment, until this carious portion has been removed; the after treatment is at best but palliative. I believe that in every serious case a line of demarcation must be produced, so that a healthy action may be re-established. I have had the most success with the use of chloride of zinc, as in my judgment it is the best stimulator of new growth of the tissue.

*Dr. Searle.* What is the strength of the solution of chloride of zinc that you are in the habit of using?

*Dr. Barrett.* I might say, all strengths, adapting it to the needs of the case in hand, varying from an extremely attenuated solution to that of saturation. In cases where I wish to produce a sloughing of the parts it is desirable to remove, I inject a saturated solution down into the pockets.

*Dr. Atkinson.* The value of every paper presented should be thoroughly apprehended by those assuming to speak upon its subject, in order that its points may be fully elucidated and brought home to the understanding of its hearers. *The* point in Dr. Harlan's paper, that did not find a lodgement in the brain of Dr. Bogue, is, that he did not see that Dr. Harlan had explicitly stated that Salivary Calculus is *one* thing, and that Pyorrhœa Alveolaris is an entirely different one. Dr. Barrett, on the other hand, evidenced his wisdom by discriminating between an escharotic and a stimulant, for there is a vast difference between them. The great majority of those using them cannot tell the difference between an escharotic, a stimulant, or a tonic, for they don't know. As I have said before a hundred times, and I repeat it again for the benefit of humanity, a solution of 20 grains of chloride of zinc in an ounce of water, is just that proportion which in its application produces the natural degree of coagulation of albuminoid substances as found in the fecundated egg, which is the forerunner of the fibrillation of the tissues.

Elixir vitriol and aqua regia, diluted with water, make the best application. Where there are no deposits of the several varieties mentioned by the learned gentleman in his paper, there should be no interference with instruments, other than the point of the syringe. It is not philosophical to use tinct. of myrrh, as water will cause the precipitation of the myrrh from its alcoholic solution.

*Dr. Shephard.* I have had a case in point which I have not hitherto reported. A physician brought me a patient tainted with specific disease, and the long course of mercurial treatment to which he had been subjected had produced long-continued and aggravated ptyalism. Upon examining the mouth I discovered pronounced Pyorrhœa Alveolaris. I told the physician that I certainly could help the patient. Pus could be expressed from the free margins of the gums in all regions of the mouth, and so free was its discharge during the night that the pillow around the place the head lay would be saturated. The mercurial treatment was discontinued. I then instituted surgical treatment, thoroughly removing all deposits, using dilute sulphuric acid as the medicament.

In two weeks I returned the patient to his physician, who renewed the mercurial treatment without a recurrence of the Pyorrhœa. This disease is largely of a local character, and due largely to exciting cases, that are also local. It can be successfully treated by surgical methods alone; the therapeutical treatment need only be that of cleanliness. After the thorough removal of all accretions, systematic cleanliness should be insisted upon. A slight stimulant, such as a dilute solution of chloride of zinc, may be used with propriety. I wish to emphasize my opinion by repeating that the deposit of Pyorrhœa Alveolaris is one thing, and Salivary Calculus is an entirely different one, as the former is found everywhere, and in localities where Salivary Calculus does not deposit. I wish, with Dr. Barrett, that we could have some light upon the etiology of Pyorrhœa Alveolaris, as I have seen nothing satisfactory on the subject; and until we possess adequate knowledge in that direction, our treatment must necessarily be empirical.

*Dr. Pierce.* I have some hesitation in speaking upon this subject, for the reason that my experience is so adverse to the courses of treatment that have just been detailed by others. I, too, have had cases of Pyorrhœa Alveolaris. In the mouths of children the condition is purely local, and yields readily to local treatment. I have seen two cases in which there existed urinary calculus, and upon examining the mouth, found that upon some of the teeth there was present the characteristic deposit of Pyorrhœa. We find that preceding this disease, there is a calcification of the tubuli, a filling up of the pulp-chamber, and deposits about the necks of the teeth. This condition precedes Pyorrhœa Alveolaris, precedes the formation of pus, and precedes the breaking down of the alveolar walls.

I do not believe that the disease can be cured, and have never seen a case that has been cured, for the reason that the affection is one due to systemic causes with which we are as yet unacquainted. My treatment for several years past has been to remove the deposits, and swab out the pockets with dilute sulphuric acid. The deposits are not *the cause* of the disease, but their presence is an *exciting* cause; consequently, their removal is indicated, supplemented with an occasional application of the acid.

*Dr. Rhein.* The key-note was struck when Drs. Barrett and Shephard mentioned the lack of our knowledge upon the etiology of the disease. In many cases the exciting cause was unquestionably some irritant, the disease yielding to constitutional treatment, and when that is dropped, I am not prepared to say whether a recurrence is due to it or not. This conclusion I have drawn from cases I have seen, and from those reported by several investigators.

*Dr. Field.* As I understand, there has never been an analysis made of the Pyorrhœal deposit. Dr. Harlan told me that there has not been gathered a sufficient amount of the deposit of which to make a chemical analysis. If this is true, why is it that the gentlemen state so positively and dogmatically that the two deposits, Salivary Calculus and that of Pyorrhœa, are so very different? I cannot say, for I have made no investigation of the subject. I have seen Salivary Calculus as hard and dense as that of Pyorrhœa, and in color so much alike that it is next to impossible to distinguish.

*Dr. Odell.* The indiscriminate use of an agent so strong as aromatic sulphuric acid may work serious damage, even in the hands of the most skillful. If used too freely, it will dissolve away the tooth tissue. Chloride of zinc is, if anything, worse.

*Dr. Pierce.* If I left the impression on the audience that I use aromatic sulphuric acid indiscriminately, and give it to the patient for self-application, I wish to correct the misapprehension. I recommend the patient to partake of acid drinks, but do not trust in his hands the use of a powerful medicament.

*Dr. Allport.* When the paper just read was under discussion in the Committee, I made some remarks on the use of Eugenol in the treatment of abscess, exposed pulps, and cutaneous affections, and as Dr. Harlan has incorporporated them in his paper, it is not necessary for me to say anything, further than that I use it. Pyorrhœa Alveolaris may be, and undoubtedly is, a constitutional disease, while on the other hand, Salivary Calculus is simply a local matter, its only treatment being its removal, and the subsequent maintenance of cleanliness. Pyorrhœa is of systemic origin; in those cases it attacks where the cleanliness of the mouth is perfect, and there is no deposit. I cannot see why the disease should attack the alveolus, unless it is an expression of a disease lurking in the system. All cases of it are associated with uric troubles; therefore, the use of instruments is only to remove a local manifestation, and is but an incidental part of the treatment. There are always bacteria where there are pockets, and there are always pockets where there are bacteria. In this case, I believe that the bacteria secrete some irritant, and thus keep up, and exaggerate the disease. This being the case, and the disease itself being one of systemic origin, the Pyorrhœa is bound to recur.' Eugenol is a powerful germicide, and also an excellent stimulant. The mineral astringents and caustics are also germ-destroyers as well as stimulants. In the treatment of all these cases, when the use of any of these agents is indicated, we should use them intelligently; they should be used only when we know *why* they are, and not because Dr. This or Dr. That recommends them.

*Dr. Truman.* There is one point, at least, in Dr. Harlan's paper that I wish to endorse, and it is that portion relating to deposits upon the teeth. What *is* Pyorrhœa Alveolaris? Is it anything more than a local lesion, aggravated by systemic conditions? Then it is only alveolar abscess. What is pus? It is only an aggregation of leucocytes, or wandering cells. What are these cells? They are simply amoeba; therefore, when this is so, we have a wasting away of the tissues. We must treat the pericementum; we must get rid of the bacteria. They seem to have a different form in Pyorrhœa Alveolaris. Whatever agent is used in the treatment, it must be one capable of destroying the germs. The idea that you can cut and slash around the root is barbarous, and if we do this, we cannot expect a renewal of tissue. I have never seen *any* deposit at the end of the root. Pyorrhœa Alveolaris is, as Dr. Pierce says, an over calicification of the tooth. I cannot conceive how any one can derive any success from the use of aromatic sulphuric acid. I *do* get success with dilute sulphuric acid, and am not afraid to use it, following its application with bi-carbonate of soda. Keep up the use of a mouth-wash containining a mild solution of thymol. Sulphate of quinia is one of the very best remedies we have, as it inhibits the migration of the leucocytes."

The hour of adjournment having arrived, the motion to adjourn was put and carried.

---

SECOND DAY—TUESDAY MORNING SESSION.

President Darby called the Association to order at 11 o'clock. The minutes of last evening's session were read and approved. Miscellaneous business was announced, and the Secretary ordered to pay such bills as were due. Dr. Crouse stated that vacancies in Section 3 were filled with the names of Drs. A. O. Hunt and L. D. Shephard. On motion, Prof. Mayr, of Springfield, Mass., was accorded the privileges of the floor. A motion was made to accord Drs. Lovejoy, Andreas, Bazin, and Bourden, of Montreal, Canada, the floor. Dr. Barrett moved to extend the same privilege to Prof. Truman. Dr. Shephard called for a division of the vote, in order to distinguish between dentists of foreign countries and those native. Dr. Barrett then withdrew the nomination of Prof. Truman. The gentlemen from Canada were then extended the privileges of the floor. A telegram was read from Dr. Thos. P. Moore, of Columbus, Ga., regretting his inability to attend on account of illness in his family. The resumption of the discussion on Dr. Harlan's paper was announced.

*Dr. Shephard.* I would like to request the members speaking upon the subject to be more explicit as to just what their treatment is, and when mentioning remedies, to give their exact proportions, as such terms as dilute sulphuric acid, etc., are indefinite and unsatisfactory.

*Dr. Searle.* Why and what? To the first question, why should we use such remedies in the treatment of Pyorrhœa Alveolaris as were mentioned last night, the answer would be, "Because the disease requires it." We have not yet gone deep enough into the causes of the disease. Decalcification of the tooth and caries are synonymous terms. Pyorrhœa Alveolaris is a disease of the pericementum, set up by an irritation that may be due either to deposits, low organisms, or mechanical causes. It works itself far down between the alveolar wall and the pericementum before it manifests itself, and unless we can apply our remedies to the parts affected, we might as well throw them away.

Ptyalism is not Pyorrhœa Alveolaris, Salivary Calculus is not, inflammation is not, unless, indeed, it has progressed to a certain stage. Now, where does Pyorrhœa begin? At a certain stage of the process of inflammation, where the pericementum itself becomes involved. In order to treat it successfully, remove all foreign matter of whatsoever nature; go down to that point where the healthy and diseased tissues meet. We must recognise it as a disease that begins in the living tissue.

We all agree that delicate instruments are necessary to the thorough removal of deposits, to the cleansing out of diseased tissue, in order to give the healthy parts a chance to live. Will you use remedies? After the surgical part of the treatment is complete, treat the wound as a wound. In some cases it is a germ disease, requiring a dressing that will destroy low organisms and also antisept. The best dressing with which I am acquainted is strong, pure carbolic acid, or some other coagulator, applied far down to the union of the healthy and diseased tissue; it must be carried down to the seat of the disease. Let it form a Lister dressing by coagulating the albumen in the tissues. What else can you do? If the teeth could be kept absolutely at rest, the healing process would take place much more rapidly. The coat of coagulated albumen serves to keep out extraneous matter, and also prevents the advent of germs, which will re-form if means are not adopted to prevent such an occurrence. After the operation I have just described, *let the parts alone*, just as an amputation is let alone, both for the sake of the comfort of the patient, and to give nature an opportunity to repair damages. External friction serves one or two good purposes: First, the removal of pulpy substances that may accumulate under the margins of the gum, and it also forces out pus; second, it produces stimulation. For this purpose the brush may be used, as it also carries away any remaining particles of matter. The mouth should be washed with *plenty* of water—flood it out, for water is a good cleanser. If desirable, some medicament may be added.

*Dr. Stockton.* Last evening some gentleman said that Pyorrhœa was simply a matter of deposits upon the teeth; another one contradicted it; another one said that a concentrated solution of chloride of zinc would cure the disease; another one said that there was no such thing as a saturated solution. Some gentlemen said that the disease is amenable to treatment, while on the other hand, Dr. Pierce says it is incurable. Dr. Harlan has possibly started at the wrong end, and, as Dr. Barrett says, if Dr. Harlan can give us its etiology, I say let our beloved brother go on. If we cannot get any light upon this point, we cannot hope to treat the disease with any degree of intelligence or success.

*Dr. R. Finley Hunt.* The investigation of the etiology, pathology, and therapeutics of Pyorrhœa must be made more thorough. All local diseases are manifestations of constitutional disturbances. We must begin at the beginning, for by so doing we cannot go amiss, and will get the benefit of constitutional treatment. Those races of men that live according to nature, possess much better teeth than we, who surround ourselves with the artificialities of civilization. We must take into consideration the influences of heredity and disposition. It is a maxim in medicine, that if the cause of a disease is removed, the disease disappears. Medicine itself does not cure. A gentleman, in his remarks last evening, attributed Pyorrhœa Alveolaris to diseases of the urinary organization, and on that point my view is that both are the result of systemic conditions, and not one the cause of the other. I have nothing to offer as to treatment that would be instructive.

*Dr. Searle.* My remarks were based upon my experience in those cases where I was unable to combat the disease until I adopted the use of carbolic acid; the disease yielded kindly to this treatment, and the patients have remained well for years.

*Dr. Pierce.* When I spoke of urinary calculus last night, I did not mean to convey the impression that Pyorrhœa Alveolaris is due to urinary calculus, but that both are due to systemic conditions. There are conditions of the mouth and teeth that we term Pyorrhœa Alveolaris, which may be due to local causes alone, and when these are removed, a cure is effected. But when it is due to systemic conditions, our efforts will avail nothing until those conditions are corrected. Patients often tell us that their parents and grandparents lost their teeth in the same manner, and this fact seems to prove a hereditary tendency.

*Dr. Watkins.* I have observed that the discussion of this subject this year is different from that of last year, and also that in the year past progress has been made. In the year to come, I think we may expect great things from our progressive brethren. I have thought that I have succeeded in curing Pyorrhœa. I had a case in which but one tooth was affected, and it was badly pushed out of place. I found a little deposit half way up the root. This I removed, and also cut away the border of the alveolus, and applied aromatic sulphuric acid, full strength. I then tied the tooth with platinum wire. At the end of three months all was healthy with the exception of a slight deposit on the mesial surface, which I had probably overlooked. This I then removed. After the lapse of three years there has been no recurrence of the disease around that tooth, though others had become affected, and are now under treatment.

*Dr. Harlan.* Last evening a gentleman wished to know how to distinguish between salivary calculus and serumal or sanguinary deposits. There is a vast difference in the physical appearances of them. In closely examining salivary deposits, it will be found that the pericementum is not detached beyond the line of the deposit in any event, it being impossible to lift the pericementum beyond this line. Whatever may be the initiation of Pyorrhœa Alveolaris, mechanical, chemical, or traumatic, it has its beginning at the gingival margin. The ligament surrounding the neck of the tooth must first be separated; if you have a nidus, it is easy to see how the deposit may accumulate. It is believed in connection with this disease that micro-organisms are present, and manufacture a solvent that destroys the tissues. This would indicate that remedies used in course of treatment must be directed to their destruction. It is necessary to re-inject to destroy the germs as they develop, for the reason that it is only the fully developed germs that are destroyed, and not the spore from which they develop.

The theory that it is micro-organisms that manufacture a soluble ferment was projected by Dr. Black, of Jacksonville, Ills. Should any of the teeth be loose, they should be wired to steady them and make them useful to the patient. When such gentlemen as Dr. Pierce take the floor and state that it is impossible to cure a case of Pyorrhœa Alveolaris which arises from constitutional causes, and that his treatment is limited to one remedy, and that it is impossible to get at the diseased spot, I don't wonder that it is impossible for him to cure a case. How can a cure be accomplished unless the proper instruments and remedies are provided with which to do the work as it should be done? It is no criticism upon a method unless this has been done, and its provisions thoroughly tested in practice.

*Dr. Brown.* Two positive statements have been made concerning this disease—that it *can* be and that it *cannot* be cured. It will take seven generations to cure the disease, to eradicate it from the system, as it is in the family blood, handed down from parent to child. Treat and treat, and keep on treating, even though the disease recurs, as it is bound to do, you cannot wipe it out of a family in three or four generations.

*Dr. Shephard.* From my observation, it is possible to wipe it out in *one* generation. We should all bear in mind that, so far as the future goes, it is in the province of the dentist to eradicate it. In its incipiency, it *is* curable. Taken in its early stage, there will be no loss of tissue, and the health of the tissues can be secured in every case. The hope of the future lies in our belief of this truth, and spreading it among our patients, in order to induce them to bring their children early in life, that they may be protected by such measures as will prevent the occurrence of serious damage later in life. As Dr. Pierce said, *tendency* only is hereditary, and when taken early, treatment can be instituted before injury results to the patient.

The meaning of the term Pyorrhœa Alveolaris is pus-flowing —nothing else than that. As we use the term, we include the loss of soft tissue and bone, but strictly speaking they are not included. There is no term by which the disease may be fully expressed in the name. Pyorrhœa Alveolaris, if I understand the term aright, is simply the symptom of a disease that has its cause *somewhere*, but is not the disease itself. I wish to emphasize this point for home guidance, and that is, that *the children must be brought to us from the time they are six or seven years of age, and that we must direct our attention to the rising generation*, if we hope to secure anything like success in the treatment."

Dr. Field moved that the subject be passed. Dr. Atkinson said that if we care anything for advancement, it were far better that we tarry where we need the most enlightenment, and that he was in favor of the discussion continuing until the subject was thoroughly elaborated. Dr. Kingsley said that if we could not learn anything about the disease by this time from what had been said by the speakers, we would not learn any more if the discussion occupied the whole meeting of the Association, and that he favored passing the subject. Dr. Taft moved to amend by continuing the subject for thirty minutes. Dr. Crouse moved to limit each speech to five minutes; lost. Dr. Allport moved to amend Dr. Taft's amendment by continuing the discussion for one hour.

Dr. Crouse moved to lay all motions on the table, this was put and as there was a disagreement the discussion proceeded.

*Dr. Atkinson.* During the discussion on Pyorrhœa Alveolaris, I have heard a good deal of sense and a great deal of nonsense. The gentlemen have defined disease in the plural, and we have got into a hotch-potch in consequence. We must get fixed in the mind as to what disease a disease is, and whether or no it can be eliminated from the system. If you can remove a cause, its effect ceases. Every phlegmonous abscess is self-limiting, provided it is let alone. A pustule can be broken up by forcing away the pus. Every alveolar abscess would cure itself, spontaneously, if there were no foreign influence at work. The recurring cases are those that were not healed up from the bottom, the closing being but temporary and retaining oxygen enough to preserve the germs alive. When you have removed that which is inimical to the health of the organization, the organization let alone, will heal itself. My pet remedy for removing and preventing the germination of bacteria is a strong solution of salicylic acid in alcohol; this produces a coagulation of the coagulable albuminoids and forms a benign pellicle that limits the territory and thus prevents the recurrence of the bacteria in their virulent form. When we have carefully, prayerfully, and religiously studied and fully investigated the original causes of disease, then, and then only, will we become the divine healers of humanity's infirmities, to which we aspire.

A motion to pass the subject was now carried.

Section 7 was called and Chairman Barrett reported a paper incidental to the subject of physiology. (Owing to Dr. Barrett's very rapid delivery, it was impossible to make an intelligent synopsis of it. Rep.) Preceding the reading of another paper on Etiology, Dr. Barrett passed around test tubes containing original cultures of fermentive fungi, made by Dr. Miller, of Berlin, himself, who has just made a flying visit to the United States. Dr. Barrett said that the morphology of the germs could not be determined under the microscope, and that the germs are not all alike and produce different effects upon the gelatin solution in which they are cultivated. The paper was now read, giving an account of Dr. Miller's experiments with deductions therefrom. (It will be published in the "REGISTER" as soon as a copy can be obtained. Rep.)

*Dr. Spaulding.* Has Dr. Miller taken the germs from caries artificially produced?

*Dr. Barrett.* Dr. Miller has produced caries artificially, by introducing normal dentine into fermentive solutions where the germs could be fully developed.

*Prof. Mayr.* A good cause is often hurt by an apparent conflict of opinions among those who are fighting for it. There are points of difference existing between Dr. Miller and the earlier advocates of the septic theory of decay, in this country. I would like to know just what is meant by the term "decay," as I think all the dispute is upon that point. Is it merely a different form of abnormal dentine, or is it a new production? When we have an abscess on the hand, though it may spread over considerable territory, the actual line of disease is not $\frac{1}{1000}$ part of an inch thick.

And not all that was done in these investigations was done by Dr. Miller, the *New England Journal* was even started with the express purpose to defend the views about the germ theory as held by the club of dentists at Springfield. Dr. Miller seems to have labored in the wrong direction; he considers as decay what was only the corpse of decay. Where is the decay? Not in the outer spongy mass, nor in the healthy dentine, but in that extremely narrow border where the process is going on; it may not be $\frac{1}{100}$ part of an inch thick; this border I tried to investigate. Dr. Miller was more active in the outer dead mass about which there is no dispute. If we have an abscess the seat of it is not in the pus nor in the sore tissue around, but in that small layer which produces the pus-cells; if we want to know anything about the abscess, this layer becomes our most important object of investigation. Let us be clear about things.

*Dr. Atkinson.* I rejoice with exceeding great joy that we have at last got to a definite point and that is, "what is decay." Our friend who has just spoken has given it to us. We must have one term to mean one thing in order to deliver ourselves from our entanglements. The track of the game makes but a sorry dinner and it is just to such feasts we have been invited, in the theories provided for our discussion. I wish to thank Prof. Mayr from the depths of my affection for saying that pathology is but perverted physiology. Pathology is not death. The decay of a tooth is but the reversal of the life currents that builded the tooth. If decay is the reversal of a current, what is that current? It is a nutrient current supplying the growing and life-giving power to the tissue. The typal order of enamel is but a modified type of dentine. Prof. Mayr did not mean decalcification when he said it. When a candle is melted, is it detallowized? I will give my friend Mayr a Delmonico dinner for saying that in the particle of matter in the tooth that has been subjected to the decaying agent the lime salts were in no way reduced in their quantity. The nutrient currents are so fine, so delicate, so *spirituelle*, that the ordinary mind, with its gross perceptions, cannot see them until they are converted into the primitive fibrillation of the embryonic organism. Let us encourage these men who are making these investigations, until they illuminate the darkness in which we are groping, and elaborate the truth, and write the vision, and make it plain, so that he who runs may read.

*Dr. Spaulding.* I have long held the opinion that there is no pathological science that is not preceded by physiology. There is no pathology *per se.* The text-books are full of descriptions of pathological processes and growths, while really there are no such things. As Dr. Atkinson says, a pathological process is but a perversion of a nutrient process.

*Dr. Barrett.* Mr. President, we did not come here to study ghosts, but to consider material things. Let us get down to a cash basis. The question concerning us is not the *track* of the game, but the game itself. Up to within a short time we have not had anything definite; so let us get down to demonstration, to the actual thing itself, and see how best we can combat the disease we elect to treat. Until a late period we have had no exact idea of the microbes present in dental caries. The idea heretofore has been that bacteria is a raging, rampant animal, that burrows its way along, and greedily devours all the tissues with which it comes in contact. The fungi of Dr. Miller, I would have you understand, are of an entirely different character. This fungus is simply and purely a *ferment.* Fermentive organisms do not exist in conjunction with putrefactive processes. Dr. Miller is an American, and claims to be an American in all his ways, and has asked me to become his mouthpiece in this country. He says: "I have been expatriated from my own country, and I have no desire to make a reputation abroad." All his papers and experiments have been made public in the United States. He has no connection with the German schools and methods of investigation, and has no desire to have any. We all recognize and appreciate Prof. Mayr and the work he has done. *He* has made *one* experiment, *very* carefully and in the solitude of his laboratory, while Dr. Miller has made fifty, and shown them to Dr. Koch and all the other investigators. In the mouth we have heat, moisture, and sugar, all favorable for fermentation. The first step is the decalcification of the enamel; next a slight decalcification of a point of dentine. This becomes spongy, and absorbs the fermentive fluids in which the germ develops. The micro-organisms secrete or form lactic acid, which decalcifies the dentine and leaves the *debris* in condition for the further development of the fermentive process. This, in rough terms, is an outline of Dr. Miller's theory of decay, and I am only sorry that he is not present to offer statements himself in furtherance of his idea. The discovery of the fermentive fungi is a *great* step in advance, and it is our duty to carry out the investigation to its logical conclusion.

*Dr. Abbott.* As Dr. Barrett says, we are here to deal with actualities. Is there nothing *beyond* what we have already seen? Have we not under the microscope been discovering *ghosts* for the past twenty or thirty years? My friend here, large as he is, was once a ghost, and a very small one, too.

*Dr Barrett* (*soto voce*). I was not worth studying then.

*Dr. Abbott.* Is it not possible that the power of the microscope may yet be indefinitely increased? In the dentine, one-third of it is organic, and this constitutes a frame, in which is deposited the lime salts. We have never claimed that there are no organisms present; they live upon the organic portion and secreting lactic acid, decalcify new portions of dentine, devour the organic portion, and so on. The organic portion is very susceptible to irritation, and if so, is equally susceptible to inflammation; it does not seem possible to account for it in any other way. My idea of caries is, that the deeper portion of it is the same as the absorption of the roots of the deciduous teeth. Dr. Miller's own experiments show that the micro-organisms do not penetrate deeply into the tooth.

*Dr. Atkinson.* They say that enamel is decayed by acids formed within the mouth, though teeth can be destroyed by acids introduced from without."

A special order of business was now announced, being the apportionment of names to the several sections. Adjourned till the evening.

At 8 o'clock Dr. Williams read a paper upon the development of the enamel, profusely illustrated by photo-micrographs projected upon a screen by the stereopticon. At the conclusion of the paper it was announced that a serious accident had happened to the little son of Dr. Clifton, of Waco, Texas, and after the Association had taken action in the matter, as reported in the September number of the REGISTER, it adjourned till next morning.

---

THIRD DAY—MORNING SESSION.

The Association was called to order by President Darby at 10 o'clock. The minutes of the second day's morning session were read and approved, there being no objection offered. The minutes of the evening session were also read and approved. Dr. Ambler, Chairman of the Committee on Resolutions relating to the death of Prof. Buckingham, reported the following:

WHEREAS, Since the last meeting of this body, the All-Wise Author of our being has, in his inscrutable wisdom, taken to himself our highly-esteemed friend and professional brother and co-laborer, Dr. Thomas L. Buckingham; and

WHEREAS, Not only this Association, but the entire profession of this and other countries will have suffered by this dispensation an irreparable loss; therefore,

*Resolved*, That we deem it fit and proper to give expression to our high appreciation of his personal worth, in that one, who was so essential to the true interest of the profession, should have been summoned to higher councils. Yet, while bowing to the will of Him who doeth all things well, we cannot refrain from giving expression to our feelings of sorrow and regret that he should have been removed from among us.

*Resolved*, That we will treasure up his past councils and profit therefrom.

*Resolved*, That these resolutions be placed on a memorial page of the proceedings of this body, and copies sent to the family of the deceased; also, to the dental journals, and that a photographic portrait be placed in the Secretary's minutes.

It was moved and seconded that the resolutions be adopted. Remarks being in order, Drs. Stockton, Taft, Allen, Frederichs, Shephard, and Searle, all had something to recall concerning the life and works of the deceased Professor. The resolutions were then adopted unanimously by a rising vote.

Dr. Atkinson read a letter from Dr. Bodecker, who is in Europe, regarding Dr. Herbst's method of filling teeth, and he expects to bring to the United States about one hundred teeth, demonstrating the applicability of the method. A motion was carried reinstating Dr. F. Y. Clark, of Saratoga, to membership. A motion was carried to allow Dr. Crowell to make some remarks on a proposed "Dentists' Protective Union." He stated that several suits had been brought against dentists by the "American Tooth Crown Co." Richmond got an injunction against several dentists on a technicality, and in his answer said that when he swore and subscribed to the papers, he did not know their contents. Some time further was spent on this matter, without much elucidation of its intricacies.

Dr. Barrett's paper, under Section 7, was called up for renewed discussion.

*Dr. Pierce.* I am very sorry that Dr. Miller is not here to state his theory for himself, as I am under the impression that Dr. Barrett has not fully appreciated Dr. Miller's exact standing. I have some extracts here, which I will read:

"Although I have now, as I think will be granted, established upon a sure basis a fact, that caries of the teeth may result directly from the action of acid producing fungi in the presence of fermentable carbo-hydrates, *the conclusion would hardly be justifiable that by keeping the mouth constantly and perfectly free from all fermentable substances, or by repeated application of ant-acids or antiseptics to all parts of the teeth, or by all these means together, we could ever banish dental caries from the oral cavity. A most powerful influence, which we do not understand, is exerted by the nutritive processes in the teeth themselves.*" * * "I am assured by men who have grown old in the practice of dentistry, that mouths which have long been under their observation, and which practically have been completely free from caries for years, at once, on account of some sudden change of health, show a general breaking down, or crumbling of the teeth, *en masse*, in the space of a few weeks." "We have here a cause which lies without the domain of both bacteria and acids."

I am deeply interested in the subject, and can strengthen the conclusions outside of the mouth. Those who have ever looked into the subject of potato rot, or other vegetable disease, whenever it has been universal, know that it has been due to some defect in the growth of the potato, which causes them to rot in the cellars where they are stored. The germs that produce the potato rot are present every year, but the circumstances must all be favorable for their development. When the atmosphere is damp and foggy for some time, farmers say, "My wheat will rot this year." This, too, is an illustration of favorable circumstances developing the germs that are always present.

These facts serve in a measure to illustrate the processes occurring in the mouth. In the broken down tissue in a cavity of decay, the germs are present. In many cases, even when the decay has been rapid, it sometimes ceases spontaneously, and without the interference of the dentist, for, though the germs are still present, the circumstances for their development and activity are no longer favorable.

*Dr. Barrett.* Dr. Pierce does not regret more keenly than I do, that Dr. Miller is not present with us, to speak for himself, as I acknowledge that I am unable to present his theories as ably as he would do it himself. Dr. Pierce does not appear to understand the nature of the theory promulgated by Dr. Miller. The germs described by him (Miller) are not bacteria, as commonly supposed, but are fermentive fungi, and assist in the process of decay, independently of bacteria. Dr. Miller's theory is not that micro-organisms are scavengers feeding upon the products of decay."

(The discussion was here interrupted by Mr. Cowan, President of the Corporation of Saratoga, who stated that the driver, who had injured Dr. Clifton's boy, would be apprehended and prosecuted for his criminality.)

*Dr. Atkinson.* It is well to discriminate just how far we have gone in this matter of striving to get an idea of the etiology of dental caries. The micro-organisms, about which so much has been said, are not the initiation of the process, by any means. The reversal of the nutrient current is the initiative cause, the micro-organisms being merely incidental.

*Dr. Pierce.* I used Dr. Miller's own words, not my own.

*Dr. Searle.* The lime-salts and basic substance are chemically united, and united by vital force. This combination, under certain circumstances of ill health, is taken down and disintegrated. Prof. Mayr has never recognized any vital power, any ghost-power, nor power of micro-organisms. Fungi are micro-organisms belonging to the vegetable kingdom, and the term "bacteria" covers all kinds of these organisms, whether of the vegetable or animal kingdom. Am I right?

*Dr. Barrett.* Yes, materially.

*Dr. Searle.* Then why make the distinction you do?

*Dr. Barrett.* I didn't.

*Dr. Searle.* I thought you did.

*Dr. Clarke.* I am sometimes of the opinion, and have almost come to the conclusion that, I have lived a worthless life, so much is there to learn, and so little have I done. I hesitate to get up and speak, when I see, meeting after meeting, the members whitewash each other for opinion's sake. The first ever known on this subject in this country was contained in a paper of mine, on bacteria, published some fifteen or twenty years ago, and in one or two papers since. I did not say much, because I did not know much. I think it is about time to stop keeping in the background the names of such investigators as Searle, Stockton, Mayr, and others, who have worked so hard in this direction. I must disagree, in toto, with Dr. Barrett. He brings with him these tubes he has just passed around, filled with gelatine solution sterilized. Sterilized by what? How has Dr. Miller excluded the air which we know to be 50 per centum organic, by these corks of cotton at the open end? If you make any distinction between fungi and bacteria, you must exclude the whole theory entirely. Fungi can only produce fungi and bacilli can only produce bacilli. I consider the whole thing a mooted question. The result of my own personal investigations so far is, that bacteria are the factors and that they produce caries by absorbing the bioplasm in the mesh-work of the dentine, leaving the lime salts to disintegrate. I contend further that there are life cells and death cells, continually fighting for supremacy. For the sake of argument we will call the bacteria, death cells, and if the person is a little careless the death cells will prevail.

*Dr. Daball.* Dr. Miller was in Buffalo a little while ago and after a dinner tendered him, he made a little exposition of his theory. One charm of Dr. Williams' paper was that every proposition he made he proved step by step, each succeeding one proving positively the one preceding. So it is with Dr. Miller. I asked him, "Dr. Miller, how do you know you are right in this matter?" He replied that he was not only fully satisfied that fungi are the cause of caries, but there is proof of his theory, he had produced genuine caries in a tooth out of the mouth. When he had succeeded in procuring a pure culture, he failed in producing the caries, having provided nothing for the fungi to feed upon, but when he furnished them with sugar as food the caries made its appearance. Dr. Miller is the only man who has ever succeeded in producing caries out of the mouth, and when a man can do *that*, I think he is on the right track.

*Dr. Clarke.* How long was the tooth out of the mouth?

*Dr. Daball.* I did not ask Dr. Miller that question.

*Dr. Clarke.* There may be no doubt that Dr. Miller is correct about his artificial caries, but he did not tell all and he don't know all. Any one may produce artificial caries in a tooth that has not been out of the mouth for a longer period than twenty-four hours. I contend that my theory as I gave it a short while ago is the correct one. In Dr. Miller's case, however, his artificial caries does not, and can not resemble in all respects the caries that takes place in the mouth as we are in the habit of seeing it.

*Dr. Daball.* We all know that a tooth immersed in vinegar or acid becomes soft. Dr. Miller claimed and demonstrated that there are many varieties of fungi, and that it is impossible to produce caries without the pure culture which is extremely difficult to obtain.

*Dr Searle.* I can not understand how Dr. Miller has succeeded in producing the identical conditions that exist in the mouth, for they must certainly be reproduced if the caries artificially produced bears a semblance to that found naturally in the mouth. Throughout the whole line of his theory as he has given it, Dr. Miller has completely ignored the work accomplished by Drs. Underwood and Mills.

*Dr. Fredericks.* He has mentioned their work and in one article in particular that I read not long ago.

*Dr Searle.* Underwood and Mills have done the work that has been done and they are entitled to the credit for it. Dr. Miller heard of their investigations and immediately began a series of rapid experiments and has poured out papers on the subject like the press of a newspaper. Though I wish to accord Dr. Miller all proper credit, yet I am compelled to say this.

*Dr. Daball.* I can't see how any one can speak of Dr. Miller in such a manner as he is a most modest and unassuming gentleman, giving every one credit for whatever he has done.

*Dr Searle.* I question Dr. Miller's ability to reproduce all the conditions under which caries takes place in the mouth.

*Dr Barrett.* I very much wish to have this subject discussed without the introduction of personalities. Mills and Underwood have not done the *kind* of work that Miller has.

A motion was now carried to pass the subject. Section 1 was called for and Dr. W. H. Trueman made a report embracing a method of pivoting teeth. Dr. Jno. Allen presented and read a paper on "Artificial Dentures," in which he said that this branch of professional work with few exceptions requires more attention than has been bestowed upon it in the last few years.

It is the province of the dentist to restore in the most scientific manner all the natural features that have been destroyed by the loss of the natural organs and his highest art is to conceal his art. He must know and be familiar with the properties and possibilities of the metals and minerals used in the work, together with the art of conforming them to his purpose.

Dr. Jerry Robinson also contributed and read a paper.

There being no remarks upon the papers read, the subject was passed. Section 2 was called and Chairman Pierce made a report detailing the action taken by the College Faculties Association, a report of which appeared in the last issue of the REGISTER.

*Dr. Spaulding.* I am very glad indeed to know that progress has been made towards elevating the standard of education, after this year at any rate. I object to the clause graduating a medical graduate after but one year's attendance at a dental college, as he cannot become qualified in that length of time. We have catered too long to the medical men, and it is time to stop it, because well qualified men have been shut out on a technicality.

*Dr. Hunt.* I am exceedingly glad to know that this good and harmonious work has been done, for I was very much afraid that differences of opinion would obstruct any advancement. Some of the things are not so well done as they might have been, but so much good has been done that we can overlook that and the future will show by experience where the defects are and they can be remedied as discovered. Let us hope and pray that the harmony between the acts of the College Faculties and National Association of Examiners will be the same as that between the several colleges, and bring about the good results this Association has desired and hoped for, so many years past.

*Dr Frederichs.* I disagree with Dr. Spaulding and am proud that the Faculties have offered the M. D.'s such inducements to become qualified dentists.

*Dr. Allport.* I desire to thank the College Faculties for the action they have taken, it comes nearer my idea of what we have all wanted than anything heretofore attempted. I believe that a medical education should be used as a basis for the further education of the dentist. Dentistry is not a profession and I believe the time will come when it will not be recognized as a profession any further than the partial study of medical subjects makes it. I believe the time is coming when every dentist *must* be medically educated before he is qualified to practice dentistry. Great stress has been laid upon a preliminary education; what is more necessary than a medical education as a foundation? The foundation of dentistry is based upon medicine and its text books and were it not for *them* where *would* we get any, as dentistry has produced none?

*Dr Abbott.* I judge from the remarks that have been made, the gentlemen all think about the same. Without anatomy where would we be from the highest round of the ladder down? Anatomy is the foundation stone of medicine, dentistry, and all that has to do with the make up of the human body. There can not be too much education so that we can not ask too much of the young, both for themselves and for our own self respect.

*Dr. Rhein.* Medical works are scientific, because they come under that department of science known as the science of medicine.

*Dr. Atkinson.* In every instance giving the medical man the honor of being a better dentist *because* he is a medical man, is a subterfuge and a falsity. Surgery as a branch of medicine is away ahead of medicine itself and beats it all hollow, because it can demonstrate what it can do. The men that know the least, respect themselves the most for they know no better (laughter). Yes! by-the-e-e *Gods*, I can point out the men.

The resolutions of the American Dental Association respecting dental education were read and Dr. Finley Hunt offered an additional resolution recognising the action of the College Faculties, which was unanimously carried: the Association then adjourned till evening.

---

### THIRD DAY—EVENING SESSION.

The session was called to order by Pres. Darby and minutes of morning session read and approved. Under the head of miscellaneous business Dr. Odell reported that Dr. Clifton's son was doing well, contrary to expectation, and all had been done that could be done to assist his father in taking care of him. The Committee on the President's address reported per Dr. J. Taft, the following amendments to the constitution:

### REPORT OF THE COMMITTEE ON PRESIDENT'S ADDRESS.

*Resolved*, That the Constitution be so changed, as to make the sections on Standing Committees, to consist of three members each. The Chairman of each committee shall be appointed for three years, the other members shall be elected, one for two years, the other for one; each to be selected with referference to their fitness for the work required of them.

These committees shall be nominated by the Executive Committee and confirmed by the Association.

We also recommend that the Constitution be so changed as to require the payment of the dues of members and delegates only when they are present at the meetings of the Association. The transactions to be sent only to members who may be present.

*Also*, That the membership of delegates be extended to three years, and that during their membership they shall be eligible to all the privileges and immunities of permanent members.

*Also*, That any permanent member who fails to attend for three years shall cease to be a member.

Dr. Fredericks moved that the report be received and the committee discharged. Dr. Shephard said no amendment to the constitution had been presented that could legally go before the Association. Dr. Kingsley moved to refer the report back to the committee to report again next morning.

*Dr. Taft.* I know that the phraseology ought to be perfected, but it is very difficult to get the committee together and the matter should be referred to somebody who will attend to the work.

Dr. Shephard said the report as it seemed to him was that it was brought before the Association in order that it might know what had been done.

This is a matter that should not be hastily thrown together as it must be carefully considered. Let the committee consider it through the coming year, and then report in print a well digested document. Dr. Frederichs withdrew his motion. Dr. Kingsley changed his motion to read that the report be recommitted and reported again next year. Dr. Shephard moved to amend by having the report printed. The motion as amended was carried. Dr. Cushing offered a resolution to change the word "Artificial," in section 7th, of the article relating to mechanical dentistry to "Prosthetic," so that it would read "Prosthetic Dentistry." Adopted by unanimous consent.

Section 3 was called for and Dr. Taft said that there was only a partial report, as the matter was in imperfect form and that he would read what there was of it, or wait till it was complete or that it could go by. On motion he read the partial report pertaining to dental literature.

*Prof. Mayr.* I have had opportunity to read all the dental journals published in the world; of the two journals published in France, a large proportion, in fact, about all the articles published, are simply translations from the American journals, the balance is nonsense. Austria with a population of 39,000,000, has no dental journal. There is one published in Spain by "permission of the Bishop," of eight pages, and contains little or nothing. There are two in England one of which consists of reports of dinners eaten by the English dentists, the other as we all know is very interesting.

*Dr. Taft.* There are three journals published in England.

*Dr. Frederichs.* Prof. Mayr slanders the French when he says their journals contain no original articles, for I have read *one* at least that I know is original; it is by Magitot.

*Prof. Mayr.* The proportion is so small that you can count on the fingers of one hand all the original articles that appear in a whole year.

Dr. Frederichs moved that the committee complete the report and that it be referred to the publication committee; carried. The subject was then passed. Section 4 was called, and Dr. Stockton took the chair, while Dr. Darby read a report, the substance of which was Herbst's method of filling teeth.

*Dr. Abbott.* I have seen teeth so filled, and they certainly look as if they will stand. The system consists essentially of packing the gold with a bulbous burnisher rotated by the engine. I doubt the possibility of myself filling teeth that way, though one does not know what he can do until he tries. I have for years used a tomato-shaped burnisher for putting on the last pieces of the filling.

*Dr. Hunt.* Is the gold that Herbst uses cohesive?

*Dr. Abbott.* My impression is that it is.

*Dr. Daball.* When Dr. Miller was here he gave a little account of his visit to Dr. Herbst, in whose office he (Miller) filled several teeth under Herbst's direction. The gold used is perfectly soft and non-cohesive. Approximal cavities are filled with the aid of a matrix fastened with shellac. If the cavity is large the gold is introduced in large masses. The filling is done very rapidly and the plug is tight and moisture proof, as tests with analine prove.

*Dr. Watkins.* When Dr. Tenney was in Germany he procured a set of the instruments and a gold that is called magnetic. He operated at the New Jersey Society, illustrating the method. Dr. Wheeler, of Albany, is perfecting a set of instruments made of stone, so as to avoid the plating of the instrument that takes place upon steel.

*Dr. McKellops.* When I first heard of the method, I sent to S. S. White and had a set made; they are not highly polished. The gold used is not cohesive. I have had different shapes made which I regard as improvements upon Dr. Herbst's.

*Dr. Barrett.* I have had a limited experience with this method of filling teeth. The Vorath gold is the softest I have ever seen and can be so consolidated as to resemble a condensed cohesive filling. Dr. Herbst's instruments are not perfectly smooth, and to remove the gold plating of them, he keeps on his table a piece of block tin with which he cleans the instruments. Though Dr. Herbst makes contour work, yet I do not think his system is adapted to it.

*Dr. A. O. Hunt.* I have had a set of instruments made, and tried some experiments out of the mouth. The difficulties are many, and I did not succeed in holding the gold in its place under the rotation of the instrument as the mass moved about from place to place. I then used a large mass to start with, and it also wabbled about for some time until it found a lodgment, after which the difficulties were mostly over. I cleansed the instruments of the adhering gold with fine emery cloth. The instrument is not continuously held upon the filling, but removed and applied in rapid succession.

*Dr. Allport.* There is both good and bad in this method. The good is that the work can be made solid, though I care nothing for that. But mark the prophecy! if it is brought into general use, so sure will we live to regret it. Even in the specimen Dr. Bodecker spoke of, though it was well packed, *yet there was a leak*, and that is the trouble with this method, it does not invariably make a secure union.

*Dr. Barker.* There is an entirely new process involved in this method. If a man can take two sheets of tin foil, and by passing his burnisher over it make it a solid, homogenous mass, I say that I have learned something new, I have learned a new principle. But to come down to the point, though the method is fascinating in its novelty and peculiarity, and is nice and all that, yet I do not believe that it will ever amount to a practical success. It seems to me that the method is nothing more nor less than a peculiar manner of burnishing.

*Dr. Clark.* I had the pleasure a few months ago of hearing a paper by Dr. Fillebrown, detailing this process of filling. I procured the gold as recommended in the paper as near as I could obtain it and made—some beautiful—failures. I think it is only history repeating itself, as a method very much like it was in use forty years ago. There is one trouble we find in the working of the method and the reason that we have failed with it is, that we are unable to get just the kind of gold that Dr. Herbst uses in making his fillings.

*Dr. Rhein.* The immense speed at which the instrument is driven develops heat enough to anneal the gold, and that, in my opinion, is the secret of the whole system.

*Dr. Watkins.* In Dr. Tenney's filling, each piece of gold was annealed before it was put in, though I did not see what was done with the first piece as it was already packed in when I saw it.

The session then adjourned.

---

LAST DAY—MORNING.

The Association was called to order by Pres. Darby, at 9:30. The minutes of last evening's session were read by the Secretary, and no objection being offered, they stood approved.

The election of officers and place of meeting were declared the order of business. After several animated speeches and three or four ballots, Minneapolis, Minnesota, was decided upon as the place of meeting for 1885.

The election of officers then followed by ballot. Dr. Crouse, of Chicago, was elected President on the first ballot, and on motion of Dr. Barrett, the election was made unanimous. Dr. Foster, of Baltimore, was elected First Vice-President; Dr. Rich, of Saratoga, Second Vice-President. It was agreed by unanimous consent that the President cast the ballot for Dr. Cushing as Recording Secretary, which was done, the same for Dr. A. W. Harlan, Corresponding Secretary and Dr. G. W. Keely, Treasurer. The executive committee elected consisted of Drs. C. N. Pierce, A. O. Hunt, L. D. Shephard.

Drs. Pierce and Abbott were appointed an Installation Committee, to conduct President-elect Dr. Crouse to the Chair, Dr. Darby formerly announcing the election, and resigning the gavel to his successor.

Drs. A. T. Smith, of Minneapolis, F. Gardner, of Chicago and H. J. McKellops were appointed as a Local Committee on arrangements.

Section 4 was passed.

Section 5 was called, and upon Dr. Rhein's requesting that the minutes be so changed as to put on record the thanks of the Association to Dr. Williams for his paper and its illustrations, or at least place on record his (Rhein's) effort to have them changed, resulted in a long heated debate on parliamentary usage, Dr. Rhein taking an appeal from the Chair, whose decision was sustained, and the agony ended.

Section 5, after some immaterial discussion, was passed, and the Association adjourned finally to re-assemble at Minneapolis, Minnesota, 1st Tuesday in August, 1885.

---

Bismarck has made his medical attendant, Dr. Schweninger, a "professor," unattached. He has no lectures to deliver, students to teach, or chair to occupy, but is simply professor of the of the anatomy, physiology, and pathology of Bismarck.—*Popular Science News.*

